# Supplementary material for: Phonon-driven spin-Floquet magneto-valleytronics in MoS2
Source: Nat Commun. 2018 Feb 12;9:638. doi: 10.1038/s41467-018-02918-5 (PMC5809408; doi:10.1038/s41467-018-02918-5)
Supplement: Supplementary file 1 — Supplementary Information [file 41467_2018_2918_MOESM1_ESM.pdf]

SUPPLEMENTARY INFORMATION

Phonon-driven spin-Floquet magneto-valleytronics in MoS_2

Dongbin Shin[1], Hannes Hübener[2], Umberto De Giovannini[2], Hosub Jin[1], Angel Rubio[2,3,4] and Noejung Park[1,2]**

[1]*Department of Physics, Ulsan National Institute of Science and Technology, UNIST-gil 50, Ulsan 44919, Korea*

[2]*Max Planck Institute for the Structure and Dynamics of Matter and Center for Free-Electron Laser Science, Luruper Chaussee 149, 22761 Hamburg, Germany*

[3]*Center for Computational Quantum Physics (CCQ), The Flatiron Institute, 162 Fifth avenue, New York, NY 10010*

[4]*Nano-Bio Spectroscopy Group, Universidad del País Vasco, 20018 San Sebastián, Spain*

Supplementary Note 1. Computational details for TDDFT calculation

The electronic states are evolved in real-time domain following the time-dependent Kohn-Sham equation which, in the system of atomic Hartree unit ($\hbar = 1$, $m_{\mathrm{e}} = 1$, and $e = 1$), can be written as follows:

$$i\frac{\partial}{\partial t}\left|\psi_{\mathrm{n},\mathbf{k}}\right\rangle = \left(\frac{1}{2}\left(-i\nabla + \frac{1}{c}\mathbf{A}_{\mathrm{ext}}(t)\right)^2 + \hat{v}_{\mathrm{atom}}(\boldsymbol{r}) + v_{\mathrm{Hxc}}[\rho(t)] + \hat{v}_{\mathrm{SOC}} + \frac{1}{2c}\hat{\boldsymbol{\sigma}}\cdot\mathbf{B}_{\mathrm{ext}}(t)\right)\left|\psi_{\mathrm{n},\mathbf{k}}\right\rangle. \quad (1)$$

The time-dependent part of the external electric field is implemented as $\mathbf{E}_{\mathrm{ext}}(t) = -\frac{1}{c}\frac{\partial}{\partial t}\mathbf{A}_{\mathrm{ext}}$ and, if any, time-independent part of the external electric field can be included in the scalar potential. The Hartree and the exchange-correlation potentials ($v_{\mathrm{Hxc}}[\rho(t)]$) are constructed from the density functional of the instantaneous charge density. The nucleus potentials are described with the pseudopotentials originated from the instantaneous position of moving atoms: $\hat{v}_{\mathrm{atom}}(\mathbf{r}) = \sum_{\lambda}\hat{v}_{\mathrm{pp}}(\mathbf{r} - \mathbf{R}_{\lambda}(t))$. Atomic positions are updated through the Ehrenfest dynamics, in which each atom is subjected to Hellmann-Feynman force. The spin-orbit coupling is implemented using the atom-centered form of approximation: $\nabla V \times \hat{\mathbf{p}} \approx \frac{\mathbf{r}}{r}\frac{\partial V}{\partial r} \times \hat{\mathbf{p}} = \frac{1}{r}\frac{\partial V}{\partial r}\hat{\mathbf{L}}$. The accuracy of the time-integration can be gauged by the consistency of the total energy. In our calculation, Crank-Nicolson type time-evolution operator was used, and the integration time step was typically chosen to be around 0.05 a.u., that is 2.42 attoseconds. For time-evolution, we used full-relativistic norm-conserving pseudopotential with 6×6×1 k-point sampling in rtp-TDDFT calculation.

Supplementary Note 2. Time-propagation using the 2×2 model Hamiltonian for the case of a linearly polarized phonon

In the text, we introduced the 2×2 model Hamiltonian for the case of the linearly polarized phonon in y-direction:

$$\hat{H}(t) = \varepsilon_0 \hat{\sigma}_z + \varepsilon_{ph} \hat{\sigma}_y \sin(\omega_{ph} t) . \quad (2)$$

To actually calculate the time propagation, we used the Szuki-Trotter type split form within the 2nd order:

$$\exp\left(-\frac{i}{\hbar}\hat{H}(t)\Delta t\right) \approx \exp\left(-\frac{i}{\hbar}\varepsilon_{ph}\hat{\sigma}_y\frac{\Delta t}{2}\right)\exp\left(-\frac{i}{\hbar}\varepsilon_0\hat{\sigma}_z\Delta t\right)\exp\left(-\frac{i}{\hbar}\varepsilon_{ph}\hat{\sigma}_y\frac{\Delta t}{2}\right), \quad (3)$$

where the component can be transformed into the following form:

$$\begin{aligned}
\exp\left(-\frac{i}{\hbar}\varepsilon_{ph}\hat{\sigma}_x\Delta t\right) &= \begin{pmatrix} \cos\left(\varepsilon_{ph}\Delta t/\hbar\right) & -i\sin\left(\varepsilon_{ph}\Delta t/\hbar\right) \\ i\sin\left(\varepsilon_{ph}\Delta t/\hbar\right) & \cos\left(\varepsilon_{ph}\Delta t/\hbar\right) \end{pmatrix} \\
\exp\left(-\frac{i}{\hbar}\varepsilon_{ph}\hat{\sigma}_y\Delta t\right) &= \begin{pmatrix} \cos\left(\varepsilon_{ph}\Delta t/\hbar\right) & -\sin\left(\varepsilon_{ph}\Delta t/\hbar\right) \\ \sin\left(\varepsilon_{ph}\Delta t/\hbar\right) & \cos\left(\varepsilon_{ph}\Delta t/\hbar\right) \end{pmatrix} \\
\exp\left(-\frac{i}{\hbar}\varepsilon_0\hat{\sigma}_z\Delta t\right) &= \begin{pmatrix} \cos\left(\varepsilon_0\Delta t/\hbar\right) - i\sin\left(\varepsilon_0\Delta t/\hbar\right) & 0 \\ 0 & \cos\left(\varepsilon_0\Delta t/\hbar\right) + i\sin\left(\varepsilon_0\Delta t/\hbar\right) \end{pmatrix}
\end{aligned} . \quad (4)$$

To simulate the real MoS_2 material, we used $\varepsilon_0 = -1.5\ \text{meV}$ which is the half of the spin splitting at the CBM. The phonon frequency of *E*" mode is used: $\omega_{ph} = \frac{2\pi}{122}\text{fs}^{-1} = 8.19\text{THz}$. The parameter ε_{ph} depends on the amplitude of *E*" phonon mode. For Figures 1c and 3a in the main text, we used $\varepsilon_{ph} = -3\varepsilon_0$. For the time-integration, we used the time integration step of $\Delta t = 48.3\text{as}$.

Supplementary Note 3. Time-propagation with the 2×2 model Hamiltonian for the circularly polarized phonon

The model Hamiltonian for the case of the circularly polarized phonon is derived as

$$\hat{H}(t) = \varepsilon_0 \hat{\sigma}_{\mathrm{z}} + \varepsilon_{\mathrm{ph}} \left(\hat{\sigma}_{\mathrm{x}} \cos(\omega_{\mathrm{ph}} t) - \hat{\sigma}_{\mathrm{y}} \sin(\omega_{\mathrm{ph}} t) \right). \quad (5)$$

The corresponding time-dependent Schrödinger equation is written as

$$i\frac{\partial}{\partial t}\left|\Psi(t)\right\rangle = \begin{pmatrix} \varepsilon_0 & \varepsilon_{\mathrm{ph}} e^{i\omega_{\mathrm{ph}} t} \\ \varepsilon_{\mathrm{ph}} e^{-i\omega_{\mathrm{ph}} t} & -\varepsilon_0 \end{pmatrix} \left|\Psi(t)\right\rangle. \quad (6)$$

By substituting $\left|\Psi(t)\right\rangle = \begin{pmatrix} e^{-i\varepsilon_0 t} C_1(t) \\ e^{i\varepsilon_0 t} C_2(t) \end{pmatrix}$ into the Supplementary equation (6), we have

$$i\frac{\partial}{\partial t}\begin{pmatrix} C_1(t) \\ C_2(t) \end{pmatrix} = \begin{pmatrix} 0 & \varepsilon_{\mathrm{ph}} e^{2i\Delta_{\mathrm{R}} t} \\ \varepsilon_{\mathrm{ph}} e^{-2i\Delta_{\mathrm{R}} t} & 0 \end{pmatrix} \begin{pmatrix} C_1(t) \\ C_2(t) \end{pmatrix}, \text{ where } \Delta_{\mathrm{R}} = \frac{\omega_{\mathrm{ph}}}{2} + \varepsilon_0. \quad (7)$$

The two 1st order equations in the Supplementary equation (7) leads to the set of 2nd order differential equation for the coefficients: $\ddot{C}_1(t) - 2i\Delta_{\mathrm{R}}\dot{C}_1(t) + \varepsilon_{\mathrm{ph}}^2 C_1(t) = 0$ and the same one for $C_2(t)$. Using the two basis solutions of these differential equations, the state ket at time t can be determined up to two undetermined constants:

$$\left|\Psi(t)\right\rangle = \begin{pmatrix} e^{-i\varepsilon_0 t} C_1 \\ e^{i\varepsilon_0 t} C_2 \end{pmatrix} = \begin{pmatrix} e^{i\frac{\omega_{\mathrm{ph}}}{2}t}\left(a e^{i\Omega_{\mathrm{R}} t} + b e^{-i\Omega_{\mathrm{R}} t}\right) \\ -\dfrac{1}{\varepsilon_{\mathrm{ph}}} e^{-i\frac{\omega_{\mathrm{ph}}}{2}t} \left\{ (\Delta_{\mathrm{R}} + \Omega_{\mathrm{R}}) a e^{i\Omega_{\mathrm{R}} t} + (\Delta_{\mathrm{R}} - \Omega_{\mathrm{R}}) b e^{-i\Omega_{\mathrm{R}} t} \right\} \end{pmatrix}, \quad (8)$$

where $\Omega_{\mathrm{R}} = \sqrt{\Delta_{\mathrm{R}}^2 + \varepsilon_{\mathrm{ph}}^2}$.

The coefficient a and b in the Supplementary equation (8) are determined by setting the initial condition. For example, for the initial spin-down state, $\left|\Psi\left(t=0\right)\right\rangle = \begin{pmatrix} 0 \\ 1 \end{pmatrix}$, the time profile of the spinor is obtained as

$$\left|\Psi\left(t\right)\right\rangle = \begin{pmatrix} -i\dfrac{\varepsilon_{\mathrm{ph}}}{\Omega_{\mathrm{R}}} e^{i\frac{\omega_{\mathrm{ph}}}{2}t} \sin\left(\Omega_{\mathrm{R}} t\right) \\ \dfrac{1}{\Omega_{\mathrm{R}}} e^{-i\frac{\omega_{\mathrm{ph}}}{2}t} \left(i\Delta_{\mathrm{R}} \sin\left(\Omega_{\mathrm{R}} t\right) + \Omega_{\mathrm{R}} \cos\left(\Omega_{\mathrm{R}} t\right)\right) \end{pmatrix}. \quad (9)$$

Supplementary Note 4. Floquet states of the model Hamiltonian

With the Hamiltonian being periodic in time, namely $\hat{H}(t) = H(t+T)$, Floquet theorem states that a quasi-stationary state can be derived from the time-dependent Schrödinger equation. By setting $\Psi_{\alpha}\left(t\right) = e^{-i\alpha t}\Phi_{\alpha}\left(t\right)$, the Floquet eigenvalue equation can be written as

$$\left[\hat{H}\left(t\right) - i\frac{\partial}{\partial t}\right]\Phi_{\alpha}\left(t\right) = \alpha\Phi_{\alpha}\left(t\right) \text{ with } \Phi_{\alpha}\left(t\right) = \Phi_{\alpha}\left(t+T\right). \quad (10)$$

For the model Hamiltonian for the circularly polarized phonon, as is used in the Supplementary equation (5), the equation becomes

$$i\frac{\partial}{\partial t}\left|\Phi_{\alpha}\left(t\right)\right\rangle = \begin{pmatrix} \varepsilon_0 - \alpha & \varepsilon_{\mathrm{ph}} e^{i\omega_{\mathrm{ph}} t} \\ \varepsilon_{\mathrm{ph}} e^{-i\omega_{\mathrm{ph}} t} & -\varepsilon_0 - \alpha \end{pmatrix} \left|\Phi_{\alpha}\left(t\right)\right\rangle. \quad (11)$$

Substituting $\left|\Phi_{\alpha}\left(t\right)\right\rangle = \begin{pmatrix} e^{-i(\varepsilon_0 - \alpha)t} C_1 \\ e^{i(\varepsilon_0 + \alpha)t} C_2 \end{pmatrix}$ in the Supplementary equation (11), we have

$$i\frac{\partial}{\partial t}\begin{pmatrix} C_1(t) \\ C_2(t) \end{pmatrix} = \begin{pmatrix} 0 & \varepsilon_{\mathrm{ph}} e^{2i\Delta_{\mathrm{R}} t} \\ \varepsilon_{\mathrm{ph}} e^{-2i\Delta_{\mathrm{R}} t} & 0 \end{pmatrix}\begin{pmatrix} C_1(t) \\ C_2(t) \end{pmatrix}, \text{ where } \Delta_{\mathrm{R}} = \frac{\omega}{2} + \varepsilon_0 . \quad (12)$$

Note the Supplementary equation (12) is in the same as the Supplementary equation (7). Thus the Floquet eigenstate can be written in the same solution forms up to undetermined constants.

$$\left|\Phi_{\alpha}(t)\right\rangle = \begin{pmatrix} e^{i\left(\frac{\omega_{\mathrm{ph}}}{2}+\alpha\right)t}\left(a e^{i\Omega_{\mathrm{R}} t} + b e^{-i\Omega_{\mathrm{R}} t}\right) \\ -\frac{1}{\varepsilon_{\mathrm{ph}}} e^{i\left(-\frac{\omega_{\mathrm{ph}}}{2}+\alpha\right)t}\left(a\left(\Delta_{\mathrm{R}}+\Omega_{\mathrm{R}}\right)e^{i\Omega_{\mathrm{R}} t} + b\left(\Delta_{\mathrm{R}}-\Omega_{\mathrm{R}}\right)e^{-i\Omega_{\mathrm{R}} t}\right) \end{pmatrix}. \quad (13)$$

But, unlike the initial condition used for the Supplementary equation (9), here we need to apply the periodic boundary condition, namely $\Phi_{\alpha}(t) = \Phi_{\alpha}(t+T)$, which yields the secular equation of the 2×2 matrix, as follows;

$$\begin{pmatrix} 1+e^{i\frac{2\pi}{\omega_{\mathrm{ph}}}(\alpha+\Omega_{\mathrm{R}})} & 1+e^{i\frac{2\pi}{\omega_{\mathrm{ph}}}(\alpha-\Omega_{\mathrm{R}})} \\ \left(\Delta_{\mathrm{R}}+\Omega_{\mathrm{R}}\right)\left(1+e^{i\frac{2\pi}{\omega_{\mathrm{ph}}}(\alpha+\Omega_{\mathrm{R}})}\right) & \left(\Delta_{\mathrm{R}}-\Omega_{\mathrm{R}}\right)\left(1+e^{i\frac{2\pi}{\omega_{\mathrm{ph}}}(\alpha-\Omega_{\mathrm{R}})}\right) \end{pmatrix}\begin{pmatrix} a \\ b \end{pmatrix} = 0 . \quad (14)$$

From the condition of vanishing determinant, the eigenvalue and eigenvector of the Floquet equation are determined.

$$\left|\Psi_{\alpha_+}\right\rangle = e^{-i\alpha_+ t}\left|\Phi_{\alpha+}\right\rangle = \frac{e^{-i\left(\frac{\omega_{\mathrm{ph}}}{2}+\Omega_{\mathrm{R}}\right)t}}{\sqrt{2}}\begin{pmatrix} \dfrac{\varepsilon_{\mathrm{ph}} e^{i\omega_{\mathrm{ph}} t}}{\sqrt{\Omega_{\mathrm{R}}\left(\Omega_{\mathrm{R}}-\Delta_{\mathrm{R}}\right)}} \\ \sqrt{\dfrac{\left(\Omega_{\mathrm{R}}-\Delta_{\mathrm{R}}\right)}{\Omega_{\mathrm{R}}}} \end{pmatrix}, \text{ with } \alpha_+ = \frac{\omega_{\mathrm{ph}}}{2} + \Omega_{\mathrm{R}} .$$

$$\left|\Psi_{\alpha_-}\right\rangle = e^{-i\alpha_- t}\left|\Phi_{\alpha-}\right\rangle = \frac{e^{-i\left(\frac{\omega_{\mathrm{ph}}}{2}-\Omega_{\mathrm{R}}\right)t}}{\sqrt{2}}\begin{pmatrix} \dfrac{\varepsilon_{ph} e^{i\omega_{\mathrm{ph}} t}}{\sqrt{\Omega_{\mathrm{R}}\left(\Omega_{\mathrm{R}}+\Delta_{\mathrm{R}}\right)}} \\ -\sqrt{\dfrac{\left(\Omega_{\mathrm{R}}+\Delta_{\mathrm{R}}\right)}{\Omega_{\mathrm{R}}}} \end{pmatrix}, \text{ with } \alpha_- = \frac{\omega_{\mathrm{ph}}}{2} - \Omega_{\mathrm{R}} . \quad (15)$$

It should be noted that, for the circularly polarized phonon with opposite polarity, a set of different Floquet eigenstates results from the same procedure. By substituting $e^{-i\omega_{\mathrm{ph}}t}$ for $e^{i\omega_{\mathrm{ph}}t}$ the Supplementary equation (11) is written as

$$i\frac{\partial}{\partial t}\left|\Phi_{\alpha}(t)\right\rangle=\begin{pmatrix}\varepsilon_0-\alpha & \varepsilon_{\mathrm{ph}}e^{-i\omega_{\mathrm{ph}}t}\\ \varepsilon_{\mathrm{ph}}e^{i\omega_{\mathrm{ph}}t} & -\varepsilon_0-\alpha\end{pmatrix}\left|\Phi_{\alpha}(t)\right\rangle \text{ leads to}$$
$$i\frac{\partial}{\partial t}\begin{pmatrix}C_1(t)\\ C_2(t)\end{pmatrix}=\begin{pmatrix}0 & \varepsilon_{\mathrm{ph}}e^{2i\Delta_{\mathrm{L}}t}\\ \varepsilon_{\mathrm{ph}}e^{-2i\Delta_{\mathrm{L}}t} & 0\end{pmatrix}\begin{pmatrix}C_1(t)\\ C_2(t)\end{pmatrix}, \text{ where } \Delta_{\mathrm{L}}=\frac{\omega_{\mathrm{ph}}}{2}-\varepsilon_0. \quad (16)$$

We note that all the difference between the Supplementary equations (11) and (16) can be contained in the different Δ parameter (Δ_{R} versus Δ_{L}). The corresponding Floquet eigenspinors can be written in the same form as the Supplementary equation (15) in term of this redefined Δ_L. We note that the corresponding Rabi frequency should also be redefined as $\Omega_{\mathrm{L}}=\sqrt{\Delta_{\mathrm{L}}^2+\varepsilon_{\mathrm{ph}}^2}$. The spin expectation for each Floquet eigenstate, for instance the S_{z} value for the α_+ spin-Floquet state $\left\langle\Psi_{\alpha_+}(t)\middle|\hat{S}_{\mathrm{z}}\middle|\Psi_{\alpha_+}(t)\right\rangle=\hbar\Big/\left(2\sqrt{1+\varepsilon_{\mathrm{ph}}^2/\Delta^2}\right)$, can also be easily obtained once we provide an appropriate Δ value.

Supplementary Note 5. Two-level model Hamiltonian with the quantized phonon

The model Hamiltonian for the two CBM bands at K valley with the presence of the right circularly polarized E'' phonon mode can be written as;

$$\hat{H}=\varepsilon_0\left(\hat{c}_{\sigma_1}^{+}\hat{c}_{\sigma_1}-\hat{c}_{\sigma_2}^{+}\hat{c}_{\sigma_2}\right)+g\left(\hat{c}_{\sigma_1}^{+}\hat{c}_{\sigma_2}\hat{b}e^{-i\omega_{\mathrm{ph}}t}+\hat{c}_{\sigma_2}^{+}\hat{c}_{\sigma_1}\hat{b}^{+}e^{i\omega_{\mathrm{ph}}t}\right), \text{(s17)}$$

where $\hat{b}$ and $\hat{b}^{+}$ represent annihilation and creation operator of the right-circularly polarized E'' phonon, $\hat{c}$ and $\hat{c}^{+}$ represent annihilation and creation operator of an electron in the σ spin state. Here, the two CBM band can be characterized by the spin index. The electron-phonon coupling constant is defined as $g_{\sigma,\sigma'} = \left\langle \psi_{\sigma} \left| \frac{\partial V}{\partial \boldsymbol{u}(E'')} \right| \psi_{\sigma'} \right\rangle$.

The solution for the Supplementary equation (17) can be pursued as

$$i\hbar \frac{\partial}{\partial t}\left|\Psi(t)\right\rangle = \hat{H}\left|\Psi(t)\right\rangle, \text{ with } \left|\Psi(t)\right\rangle = \sum_{\mathrm{n}}\left(D_{\sigma_1,\mathrm{n}}(t)\left|\sigma_1;n\right\rangle + D_{\sigma_2,\mathrm{n}}(t)\left|\sigma_2;n\right\rangle\right). \quad (18)$$

Note the application of the Hamiltonian on each component, as follows:

$$\begin{aligned}
\hat{H}\left|\sigma_2;n\right\rangle &= -\varepsilon_0\left|\sigma_2;n\right\rangle + g\sqrt{n}e^{-i\omega_{\mathrm{ph}}t}\left|\sigma_1;n-1\right\rangle, \\
\hat{H}\left|\sigma_1;n\right\rangle &= \varepsilon_0\left|\sigma_1;n\right\rangle + g\sqrt{n+1}e^{i\omega_{\mathrm{ph}}t}\left|\sigma_2;n+1\right\rangle, \\
\hat{H}\left|\sigma_2;n+1\right\rangle &= -\varepsilon_0\left|\sigma_2;n+1\right\rangle + g\sqrt{n+1}e^{-i\omega_{\mathrm{ph}}t}\left|\sigma_1;n\right\rangle, \\
\hat{H}\left|\sigma_1;n+1\right\rangle &= \varepsilon_0\left|\sigma_1;n+1\right\rangle + g\sqrt{n+2}e^{i\omega_{\mathrm{ph}}t}\left|\sigma_2;n+2\right\rangle.
\end{aligned} \quad (19)$$

The Schrödinger equation is written as;

$$\begin{aligned}
i\frac{\partial}{\partial t}\left|\Psi\right\rangle = \ldots &+ D_{\sigma_1,\mathrm{n}}(t)\left[\varepsilon_0\left|\sigma_1;n\right\rangle + g\sqrt{n+1}e^{i\omega_{\mathrm{ph}}t}\left|\sigma_2;n+1\right\rangle\right] \\
&+ D_{\sigma_2,\mathrm{n+1}}(t)\left[-\varepsilon_0\left|\sigma_2;n+1\right\rangle + g\sqrt{n+1}e^{-i\omega_{\mathrm{ph}}t}\left|\sigma_1;n\right\rangle\right] + \ldots
\end{aligned} \quad . (20)$$

By applying $\left\langle \sigma_1, n\right|$ and $\left\langle \sigma_2, n+1\right|$ on both sides of Supplementary equation (20), the 2×2 equation for the coupled subspace can be derived as

$$i\frac{\partial}{\partial t}\begin{pmatrix} D_{\sigma_1,\mathrm{n}} \\ D_{\sigma_2,\mathrm{n+1}} \end{pmatrix} = \begin{pmatrix} \varepsilon_0 & g\sqrt{n+1}e^{i\omega_{\mathrm{ph}}t} \\ g\sqrt{n+1}e^{-i\omega_{\mathrm{ph}}t} & -\varepsilon_0 \end{pmatrix}\begin{pmatrix} D_{\sigma_1,\mathrm{n}} \\ D_{\sigma_2,\mathrm{n+1}} \end{pmatrix}. \quad (21)$$

In the equation (6) from equation (5) of the main text, we discussed that this second-quantized equation produces the same results once we substitute $\varepsilon_{\mathrm{ph}} = g\sqrt{n+1}$ in the semi-classical description.

Supplementary Note 6. Strength of the induced magnetic field with respect to the phonon occupation number

Through the comparison between the results of rtp-TDDFT and model Hamiltonian, we evaluated the dependence of the induced magnetic field on the occupation number of the E'' phonon. As shown in Supplementary Figs. 2a, 2b, and 2c, we calculated the spin trajectory with various initial kinetic energies. On the other hand, using the model Hamiltonian given in Supplementary equation (2), we calculated the same spin trajectory by varying $\varepsilon_{\mathrm{ph}}$ value with the given ε_0 and ω_{ph} values. We selected the $\varepsilon_{\mathrm{ph}}$ value which can reproduce the rtp-TDDFT spin trajectory. The results are summarized in Supplementary Fig. 2d. The horizontal axis, the kinetic energy plus the potential energy of the lattice, can be compared with the phonon energy quantum: $\hbar\omega_{\mathrm{ph}}\left(n_{\mathrm{ph}} + \frac{1}{2}\right)$. We note that zero-phonon ($n_{\mathrm{ph}} = 0$) and single-phonon ($n_{\mathrm{ph}} = 1$) produces the strength of magnetic field corresponding to $\varepsilon_{\mathrm{ph}} = 2.4\varepsilon_0$ and $\varepsilon_{\mathrm{ph}} = 4.2\varepsilon_0$, respectively.

Supplementary Note 7. Phononic circular dichroism and valley-magnetism

Here, we consider time evolution of a non-magnetic valley electronic configuration in which a spin-down and a spin-up electron are in the K and K′ valley, respectively. Under the effect of a right circularly polarized phonon, the time-dependent spinor at K (evolved from the initial spin-down state) can be resolved into two Floquet eigenstate given in Supplementary equation (15):

$$\begin{aligned} &\left|\Psi(t)\right\rangle_{\mathrm{K}} = D_1\left|\Psi_{\alpha_+}(t)\right\rangle_{\mathrm{K}} + D_2\left|\Psi_{\alpha_-}(t)\right\rangle_{\mathrm{K}}, \\ &\text{where } D_1 = \frac{\sqrt{\Omega_{\mathrm{R}} - \Delta_{\mathrm{R}}}}{\sqrt{2\Omega_{\mathrm{R}}}}\text{, and } D_2 = -\frac{\sqrt{\Omega_{\mathrm{R}} + \Delta_{\mathrm{R}}}}{\sqrt{2\Omega_{\mathrm{R}}}}. \end{aligned} \quad (22)$$

The expectation value of the z-component of the spin is

$$S_{\mathrm{K,z}}(t) = {}_{\mathrm{K}}\left\langle \Psi(t)\middle|\hat{S}_{\mathrm{z}}\middle|\Psi(t)\right\rangle_{\mathrm{K}} = -\frac{1}{2\Omega_{\mathrm{R}}^2}\left(\Delta_{\mathrm{R}}^2 + \varepsilon_{\mathrm{ph}}^2\cos 2\Omega_{\mathrm{R}}t\right). \quad (23)$$

The same procedure can be applied to the electron at K′ valley evolved from the initial spin-up state.

$$\begin{aligned} &\left|\Psi(t)\right\rangle_{\mathrm{K'}} = D_1\left|\Psi_{\alpha_+}(t)\right\rangle_{\mathrm{K'}} + D_2\left|\Psi_{\alpha_-}(t)\right\rangle_{\mathrm{K'}}, \\ &\text{where } D_1 = \frac{\sqrt{\Omega_{\mathrm{L}} - \Delta_{\mathrm{L}}}}{\sqrt{2\Omega_{\mathrm{L}}}}\left(\Omega_{\mathrm{L}} + \Delta_{\mathrm{L}}\right)\text{, and } D_2 = -\frac{\sqrt{\Omega_{\mathrm{L}} + \Delta_{\mathrm{L}}}}{\sqrt{2\Omega_{\mathrm{L}}}}\left(\Omega_{\mathrm{L}} - \Delta_{\mathrm{L}}\right), \quad (24) \\ &\text{and } S_{\mathrm{K',z}}(t) = {}_{\mathrm{K'}}\left\langle \Psi(t)\middle|\hat{S}_{\mathrm{z}}\middle|\Psi^{\uparrow}(t)\right\rangle_{\mathrm{K'}} = \frac{1}{2\Omega_{\mathrm{L}}^2}\left(\Delta_{\mathrm{L}}^2 + \varepsilon_{\mathrm{ph}}^2\cos 2\Omega_{\mathrm{L}}t\right). \end{aligned}$$

We note that the two spins evolve following the Supplementary equations (23) and (24) but with the different parameter. The time-averaged out-of-plane spin values are $\bar{S}_{\mathrm{K,z}} = -\frac{\Delta_{\mathrm{R}}{}^2}{2\Omega_{\mathrm{R}}{}^2}$ and $\bar{S}_{\mathrm{K',z}} = \frac{\Delta_{\mathrm{L}}{}^2}{2\Omega_{\mathrm{L}}{}^2}$. For instance, for the case of the zero-point ($n_{\mathrm{ph}} = 0$) phonon, using

$\omega_{\mathrm{ph}} = \dfrac{2\pi}{122}\ \mathrm{fs}^{-1} = 8.1\mathrm{THz}$ and $\varepsilon_0 = 0.011\,\mathrm{mRy}$, the value of Δ_{R} and Δ_{L} are $1.35\ \mathrm{mRy}\,\hbar^{-1}$ and $1.129\ \mathrm{mRy}\,\hbar^{-1}$, respectively. The range of $S_{\mathrm{z}}(t)$ value of spin-down at K valley is $-0.5\,\hbar \le S_{\mathrm{K,z}} \le -0.481\,\hbar$ and the range of $S_{\mathrm{z}}(t)$ value of spin-down at K′ valley is $0.473\,\hbar \le S_{\mathrm{K',z}} \le 0.5\,\hbar$. The time-averaged total spin is, as shown in Fig. 4b of the main text,

$$\boldsymbol{S}_{\mathrm{avg}} = \lim_{t\to\infty}(1/t)\int_0^t \left(\boldsymbol{S}_{\mathrm{K}}(\tau) + \boldsymbol{S}_{\mathrm{K'}}(\tau)\right) d\tau = \left(-\frac{\Delta_{\mathrm{R}}^{\ 2}}{2\Omega_{\mathrm{R}}^{\ 2}} + \frac{\Delta_{\mathrm{L}}^{\ 2}}{2\Omega_{\mathrm{L}}^{\ 2}}\right)\hat{\mathbf{z}} = (-0.0039\ \hbar)\hat{\mathbf{z}}\,. \quad (25)$$

For the $n_{\mathrm{ph}} = 1$ case, the circularly polarized E'' phonon generates $\varepsilon_{\mathrm{ph}} = 3\varepsilon_0$ in-plane magnetic field and the average of total system spin becomes $S_{\mathrm{avg}} = -0.012\ \hbar$. These time-averaged non-zero spin values correspond to $-0.0078\mu_{\mathrm{B}}$ and $-0.024\mu_{\mathrm{B}}$ for $n_{\mathrm{ph}} = 0$ and $n_{\mathrm{ph}} = 1$, respectively.

Supplementary Note 8. Spin-Floquet magneto-valleytronics in bilayers of TMDC

We extended the model Hamiltonian study of spin-Floquet magneto-valleytronics to the cases of TMDC bilayers. The E'' phonons in the bilayer have two branches, E_{u} and E_{g}, which are depicted in Supplementary Table 2a. Details of the bilayer phonon structures can be found in a recent literature.[1] We focus on the coupling of the CBM spinors to E_{u} phonon which is known to be IR-active. There are four valleys in the case of the bilayer: K and K′ in each of upper and lower layers. The spin on the K point of both the upper and lower layer experience the same in-plane magnetic field, in response to a E_{u} phonon, while the out of plane magnetic fields of them are directed oppositely. As explained in the case of monolayer, the time-reversal partners

(the spin on K′ point) in each layer are subjected to exactly opposite direction of in-plane and out-of-plane magnetic field. As a result, the spin-up states (that of K in the upper layer and that of K′ in lower layer) evolves with the left-handed Hamiltonian, whereas the spin-down states (that of K′ in the upper layer and K in the lower layer) experiences the right-handed Hamiltonian, as given in Supplementary Table 2. The dichroic behavior of the circularly polarized E_u phonon can be parametrized by the Δ parameters. For the same phonon with the opposite circular polarity, the parameters for the four valleys need to be interchanged: Δ_R to Δ_L and vice versa. The same study revealed that the E_g phonon drives the spin in the upper layer exactly opposite to those in the lower layer, keeping the time-reversal symmetry at all time. However, this uninteresting E_g mode is not IR-active, and thus a real experiment can excite only E_u avoiding E_g.

The time-averaged total spin of the bilayer WTe_2 is summarized in Supplementary Fig. 5. As a result of the interplay between the opposite spin direction between the layers and the opposite effective magnetic field between the time-reversal partners, the bilayer produces almost twice increased S_z value while the noisy in-plane component is cancelled in the time-averaged profile, as shown in Supplementary Figs. 5c and 5d.

Here, we would also like to summarize the features of the electronic structure of the bilayer. A distinct character of the bilayer (or a thicker layer) is the emergence of the indirect nature in the band gap. The VBM locates obviously on the Γ-point as a result of interlayer hybridization. On the other hand, the structure of CBM of the bilayer is not so obvious. Whether the energy minimum is on K(K′) or Σ depends on stacking configuration and also computational method. For example, a recent GGA+GW calculation reported that all 2H-polytype TMDC bilayers have its CBM minimum on Σ.[2] In the same literature, on the other

hand, it was shown that heterogeneous layers (such as $MoS_2/MoSe_2$, $MoSe_2/MoTe_2$, WSe_2/WS_2, WSe_2/WTe_2) have their CBM on K(K'). Another previous study using HSE06 hybrid functional reported that heterogeneous trilayers have their CBMs on K(K').[3] Thus, there are numerous combinations of the bilayer on which the phonon driven spin-Floquet magnetism can be realized. Even when the CBM locates on Σ, our model for the spin-Floquet valley magnetism is still valid because the spins on the CBM of Σ is as rigid as the VBM of K. To prove this, we calculated the variation of the spin direction of the CBM of K, CBM of Σ, and VBM of K, as summarized in the Supplementary Fig. 3. It shows that the spins at CBM of Σ and VBM of K remain near the equilibrium direction irrespective of the lattice displacement.

Schematic displacement	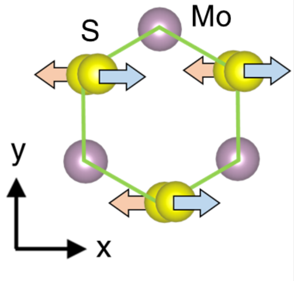	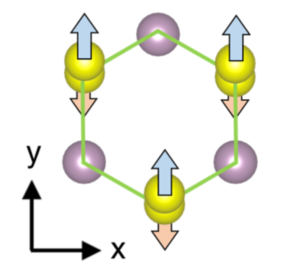	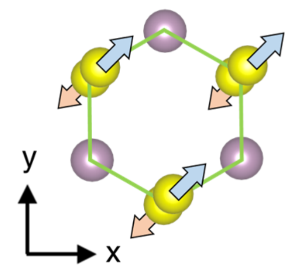
Atomic displacement	$\mathbf{d}_s = (0.1, 0.0, 0.0)$ Å	$\mathbf{d}_s = (0.0, 0.1, 0.0)$ Å	$\mathbf{d}_s = \left(\frac{0.1}{\sqrt{2}}, \frac{0.1}{\sqrt{2}}, 0.0\right)$ Å
Effective magnetic field	$B_{ph}(1.0, 0.0, 0.0)$	$B_{ph}(0.0, 1.0, 0.0)$	$B_{ph}\left(\frac{1}{\sqrt{2}}, \frac{1}{\sqrt{2}}, 0.0\right)$
CBM(K)	$\langle \mathbf{S} \rangle = (\pm 0.5, 0.0, 0.0)$	$\langle \mathbf{S} \rangle = (0.0, \pm 0.5, 0.0)$	$\langle \mathbf{S} \rangle = \left(\pm\frac{0.5}{\sqrt{2}}, \pm\frac{0.5}{\sqrt{2}}, 0.0\right)$
VBM(K)	$\langle \mathbf{S} \rangle = (0.0, 0.0, -0.5)$	$\langle \mathbf{S} \rangle = (0.0, 0.0, -0.5)$	$\langle \mathbf{S} \rangle = (0.0, 0.0, -0.5)$

Supplementary Table 1 | Spin polarization direction of CBM and VBM edge states at K valley when MoS_2 statically distorted along the eigenvector of E'' mode. The phonon eigenvector can be chosen in various in-plane directions, and in this table, we take the displacement of S atom in $\hat{x}$, $\hat{y}$, or $\frac{1}{\sqrt{2}}(\hat{x}+\hat{y})$ direction. B_{ph} is the effective magnetic field induced by the displacement along such chosen direction of the E'' phonon mode.

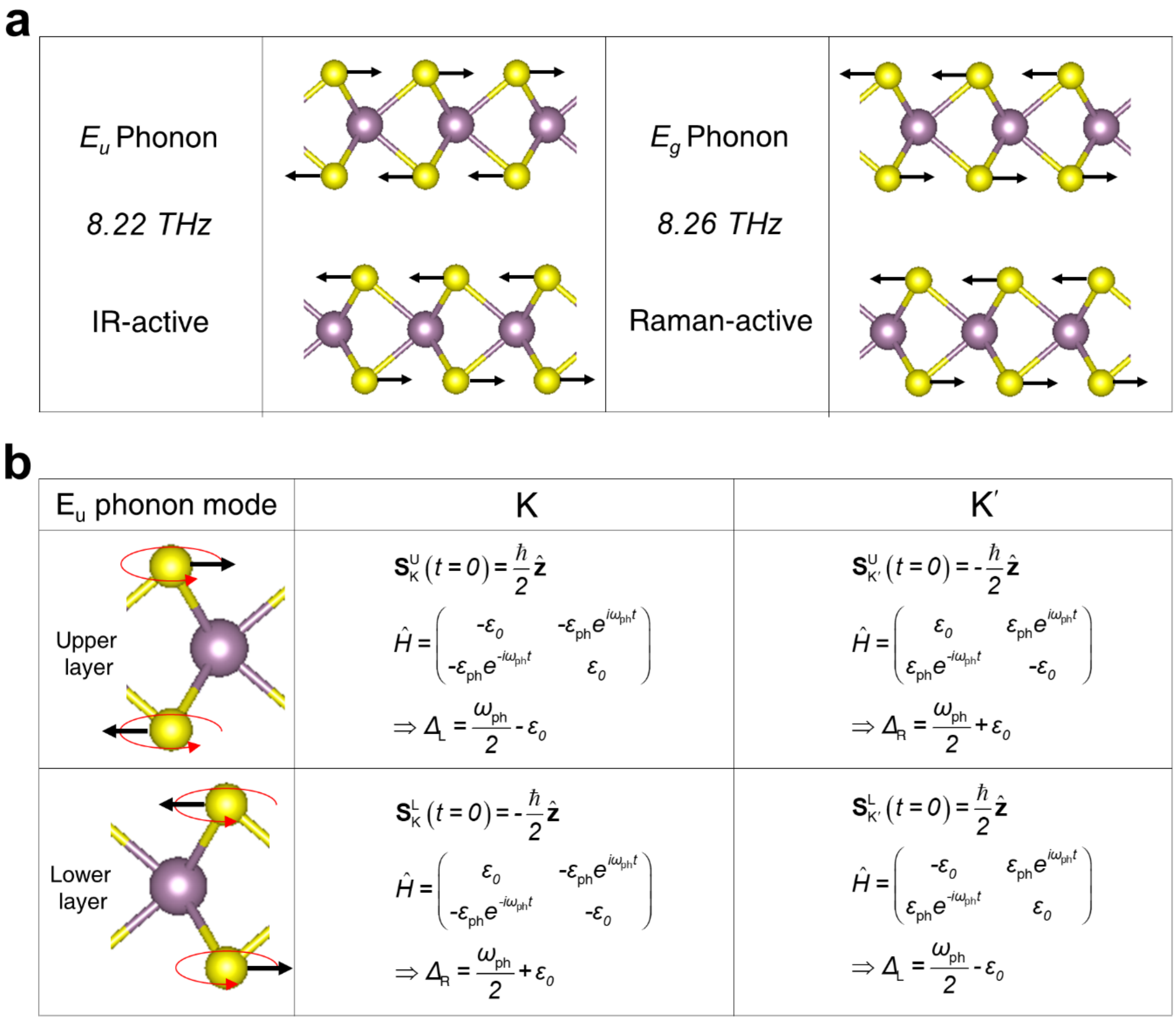

Supplementary Table 2 | Phonon-driven spin-Floquet magneto-valleytronics in TMDC bilayer. a, The phonon eigenvectors, corresponding to the *E*'' in the case of monolayer, have two branches in bilayer: E_u and E_g. **b**, The model Hamiltonian for the dichroic behavior of the spin-Floquet states of valleys with respect to a circularly polarized E_u phonon. Note that the spin-Floquet state can be described by the same form as eq. 4 in the main text, once the Δ values are defined, as given in this table.

TMDCs	$\Delta E_{VBM,spin\text{-}split}$	$\Delta E_{CBM,\ spin\text{-}split}$	E″ phonon
CrS_2	70 meV	3.6 meV	7.8 THz
$CrSe_2$	92 meV	16 meV	4.4 THz
$CrTe_2$	109 meV	21 meV	3.1 THz
MoS_2	150 meV	3 meV	8.2 THz
$MoSe_2$	188 meV	20 meV	4.8 THz
$MoTe_2$	219 meV	32 meV	3.3 THz
WS_2	440 meV	28 meV	8.5 THz
WSe_2	481 meV	34 meV	4.9 THz
WTe_2	504 meV	48 meV	3.4 THz

Supplementary Table 3 | The SOC splitting at VBM and CBM, and the *E*'' phonon frequencies of various TMDCs. To achieve a spin-resolved electron population in CBM valleys through the electrostatic doping, a larger SOC splitting is favored, and thus $MoTe_2$, WTe_2 and WSe_2 are better candidates for a realistic experiment.

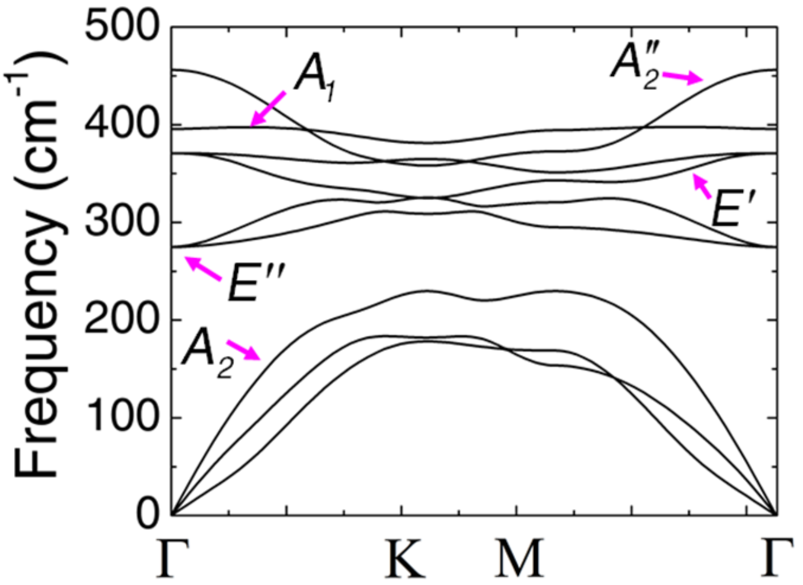

Supplementary Figure 1 | Phonon dispersion of monolayer MoS_2. Phonon structures were calculated by density functional perturbation theory.

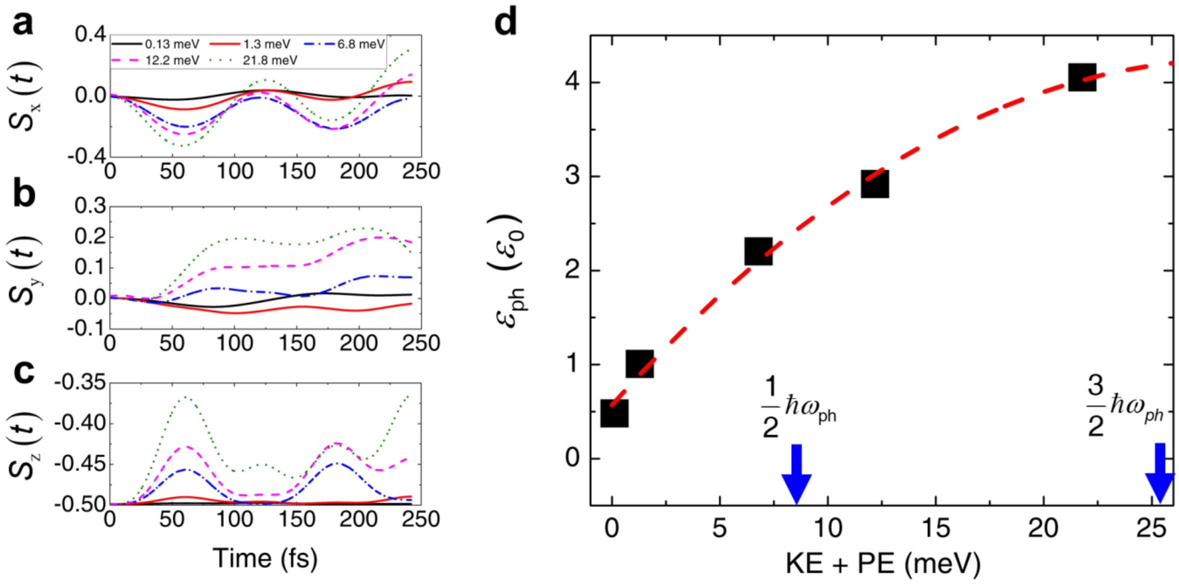

Supplementary Figure 2 | Strength of the induced magnetic field depending on number of phonons. a-c, The time profiles of Cartesian components of the spin vector with respect to lattice vibration energy. **d,** Induced magnetic field, renormalized into ε_{ph} as defined in the main text, in terms of the lattice vibration energy. The red dashed line is only guide to the eye. The phonon energy quanta are indicated by downward arrows.

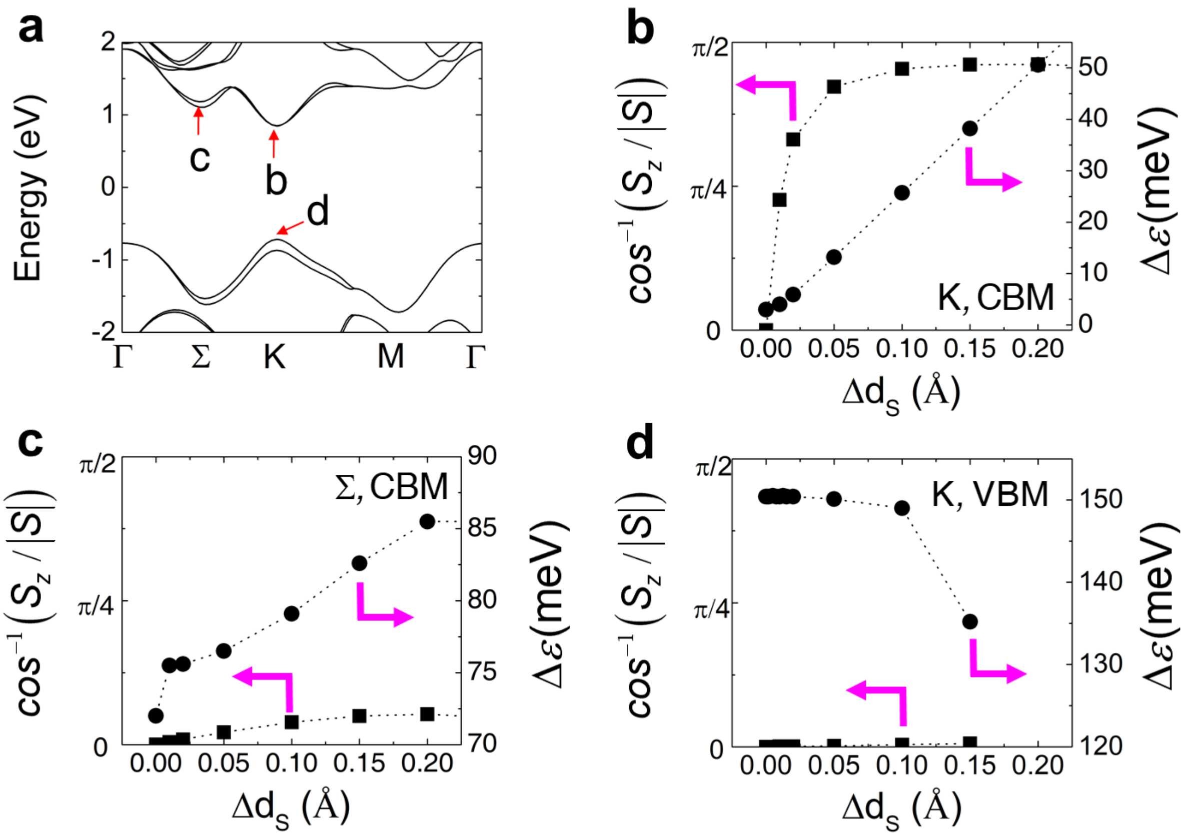

Supplementary Figure 3 | Electronic and spin configuration of monolayer MoS_2 with and without a static lattice displacement along an *E*'' phonon eigenvector. a, Band structure of the equilibrium monolayer MoS_2. **b-d**, The variations in the spin angle and the SOC splitting ($\Delta\varepsilon$) with respect to the magnitude of the displacement at (**b**) CBM of Σ point, (**c**) CBM of K point, and (**d**) VBM of K point.

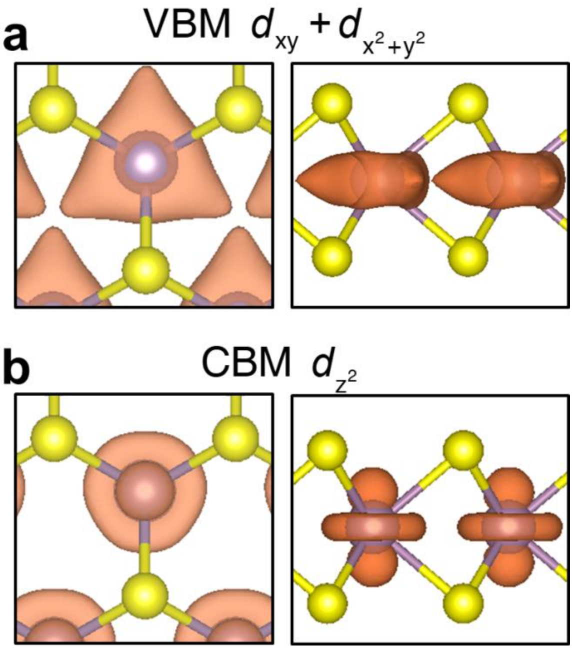

Supplementary Figure 4 | Dominant orbital character in VBM and CBM states. a-b, Orbital character of (**a**) VBM and (**b**) CBM valleys of monolayer MoS_2.

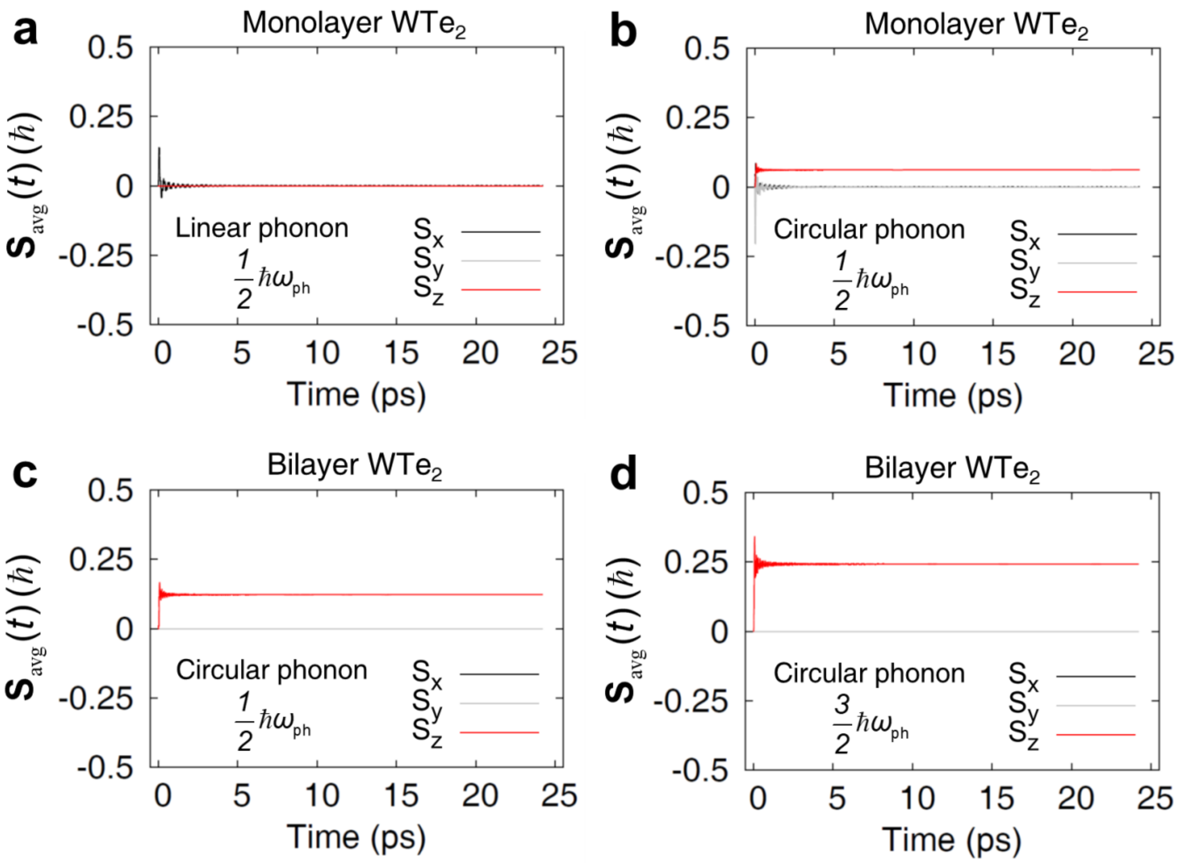

Supplementary Figure 5 | The time-averaged total spin values of the monolayer and bilayer WTe_2 with an E'' and an E_u phonon mode, respectively. a-b, The cumulative time average of the total spin in monolayer WTe_2 with (**a**) linear and (**b**) circular polarized E'' phonon. **c-d**, The same total spin of the bilayer WTe_2 with a circular polarized E_u phonon energy with (**c**) zero-point phonon ($\frac{1}{2}\hbar\omega_{ph}$) and (**b**) single phonon ($\frac{3}{2}\hbar\omega_{ph}$).

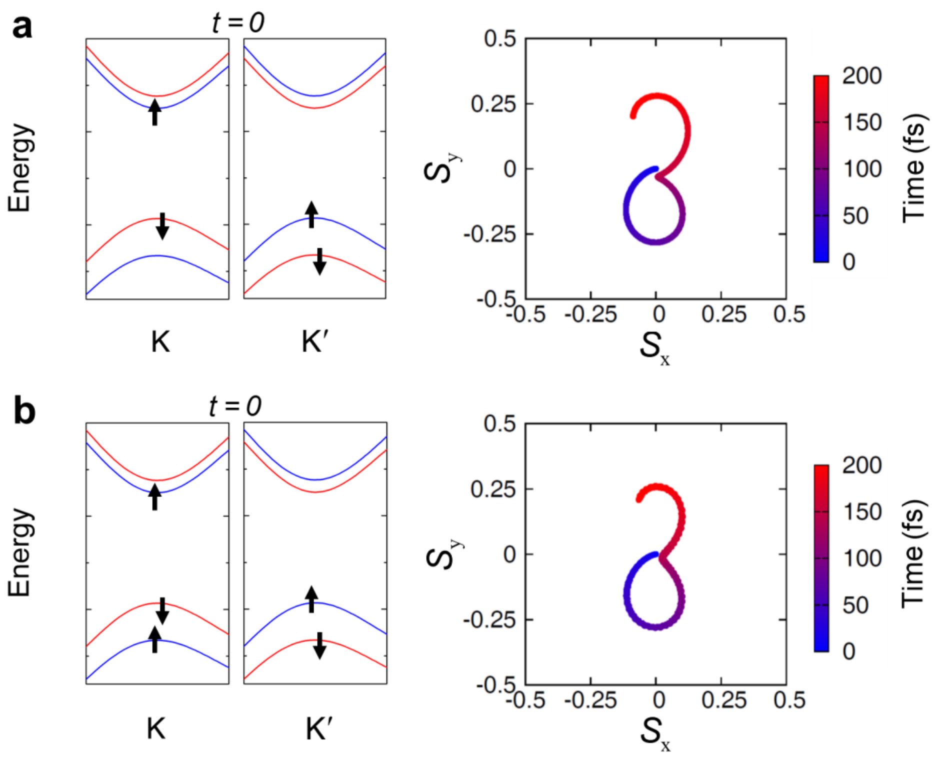

Supplementary Figure 6 | The time evolution profile of the CBM spins at K valley of the monolayer WTe_2 on the presence of a linearly polarized *E*'' phonon. a-b, The initial state was prepared through (**a**) the electron-hole excitation and (**b**) the electrostatic electron doping. The left panels in (**a**) and (**b**) depicts the initial electronic configuration. The hole spins in (**a**) remained almost rigid throughout the simulation time, and the presence of hole in the VBM has negligible effect on the dynamics of the CBM spin. In this simulation, the phonon energy is fitted to the zero-point vibration, that is, $E_{\mathrm{ph}} = \frac{1}{2}\hbar\omega_{\mathrm{ph}}$ which correspond to $\varepsilon_{\mathrm{ph}} = 0.44\varepsilon_0$, where ε_0 is the CBM splitting. The same calculation with single phonon ($E_{\mathrm{ph}} = \frac{3}{2}\hbar\omega_{\mathrm{ph}}$) led to similar result.

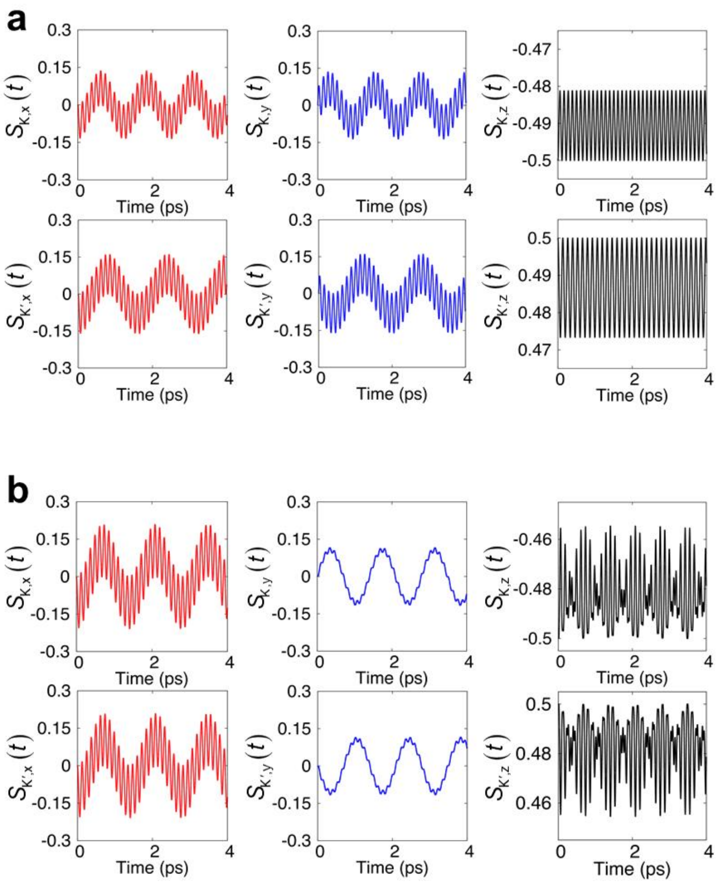

Supplementary Figure 7 | Spin-trajectory at K and K′ valley driven by *E*'' phonon mode. **a-b**, Time variation of the Cartesian components of the spin with (**a**) the circularly polarized and (**b**) the linearly polarized phonon. Upper and lower panels are for the spin-down electron at K point ($\mathbf{S}_{\mathrm{K}}(t)$) and the spin-up at K′ ($\mathbf{S}_{\mathrm{K}'}(t)$), respectively.

Supplementary Reference

1. Zhao, Y. *et al.* Interlayer Breathing and Shear Modes in Few-Trilayer MoS_2 and WSe_2. *Nano Lett.* **13**, 1007 (2013).
2. Debbichi, L., Eriksson, O. & Lebegue, S. Electronic structure of two-dimensional transition metal dichalcogenide bilayers from *ab initio* theory. *Phys. Rev. B* **89**, 205311 (2014).
3. Lu, N., Guo, H. Y., Wang, L., Wu, X. J. & Zeng, X. C. van der Waals trilayers and superlattices: modification of electronic structures of MoS_2 by intercalation. *Nanoscale* **6**, 4566 (2014).
